# Identification of Protein–Excipient Interaction Hotspots Using Computational Approaches

**DOI:** 10.3390/ijms17060853

**Published:** 2016-06-01

**Authors:** Teresa S. Barata, Cheng Zhang, Paul A. Dalby, Steve Brocchini, Mire Zloh

**Affiliations:** 1 EPSRC Centre for Innovative Manufacturing in Emergent Macromolecular Therapies, University College London, Biochemical Engineering Department, Bernard Katz Building, Gordon Street, London WC1H 0AH, UK; c.zhang.11@ucl.ac.uk (C.Z.); p.dalby@ucl.ac.uk (P.A.D.); ucnvsbr@ucl.ac.uk (S.B.); 2 UCL School of Pharmacy, Department of Pharmaceutics, 29-39 Brunswick Square, London WC1N 1AX, UK; 3 Department of Pharmacy, Pharmacology and Postgraduate Medicine, University of Hertfordshire, College Lane, Hatfield AL10 9AB, UK

**Keywords:** molecular docking, protein–excipient interactions, protein stability, molecular dynamics, Fab formulation

## Abstract

Protein formulation development relies on the selection of excipients that inhibit protein–protein interactions preventing aggregation. Empirical strategies involve screening many excipient and buffer combinations using force degradation studies. Such methods do not readily provide information on intermolecular interactions responsible for the protective effects of excipients. This study describes a molecular docking approach to screen and rank interactions allowing for the identification of protein–excipient hotspots to aid in the selection of excipients to be experimentally screened. Previously published work with *Drosophila* Su(dx) was used to develop and validate the computational methodology, which was then used to determine the formulation hotspots for Fab A33. Commonly used excipients were examined and compared to the regions in Fab A33 prone to protein–protein interactions that could lead to aggregation. This approach could provide information on a molecular level about the protective interactions of excipients in protein formulations to aid the more rational development of future formulations.

## 1. Introduction

Therapeutic proteins are potent drug substances that are used and being widely developed to address unmet clinical needs in oncology, inflammation, and chronic pain. Proteins are dynamic structures susceptible to both aggregation and unfolding processes since the tertiary structure is primarily maintained by non-covalent interactions. Excipients are used in an effort to minimise deleterious processes leading to loss of biological activity. A key challenge is to formulate clinically used proteins so that they can be stored without loss of biological activity.

Understanding protein–excipient interactions is important to optimise protein formulations. These interactions can change the surface properties of the proteins and may change their secondary and tertiary structure [1,2], which could be detrimental for protein activity. Since proteins are administered parentally, the number of excipients that are clinically approved is limited. The formulation process usually involves screening these excipients at different relative concentrations. These experiments are often challenging because of the low availability of the protein of interest during preclinical studies. Furthermore, excipient selection is often empirical or based on historical data and amino acid sequence observation, without consideration for the protein’s three-dimensional structure. Companies seek alternative strategies to overcome this limitation.

The rational development of formulations derived from optimised interactions between proteins with specific excipients that effectively contribute to their stability and efficiency would be an important step-change in the field. Currently, there are attempts to use a combination of molecular docking and dynamics in selection of excipients to improve drug solubility [3,4] or to define the protein surfaces where the excipients may bind [5,6].

Molecular docking has been extensively used in drug discovery both for the design of new drugs tailored to specific targets and to understand the interactions with known molecules that provide a scaffold for new, improved design. Still, the potential of molecular docking techniques for formulation design and optimisation has not been utilised to the same extent.

In this study, we focus on the use of molecular docking techniques to develop a computational approach that will enable informed excipient selection by identifying hotspots for their interaction on the protein surface. The novelty of this approach lies in the attempt to identify interaction surfaces where an excipient may form favourable interactions with a protein. Excipients whose predicted interaction surfaces coincide with protein–protein interaction surfaces potentially responsible for protein aggregation may be good candidates for inclusion in protein formulation. The identification of protein–excipient interaction surfaces is carried out without prior knowledge of binding sites in contrast to well established docking methods used in structure-based drug design where the ligand binding sites are known. Our approach fully considers the three-dimensional structure of a protein and provides a time-efficient approach suited to be used in early formulation stages of protein therapeutics development regardless of protein class. The development of this approach has the potential to increase our ability to rationally design protein formulations during early preclinical development.

A well-studied protein, *Drosophila* Su(dx) protein (WW34), was used to develop our computational approach to hotspot identification. Two different studies—one experimental and one computational—inspected the role of arginine (Arg) and glutamic acid (Glu) in this protein solubility. These studies were used as a reference for the development and validation of our approach.

Firstly, the effect of the combined addition of Arg and Glu to a solution of the WW domains 3 and 4 from *Drosophila* Su(dx) protein (WW34) in the reduction of intermolecular protein–protein interactions has been studied experimentally by Golovanov *et al.* [7]. In this study, the authors establish the simultaneous addition of 50 mM of Arg and Glu as a method to increase the solubility of WW34. They studied this effect with several different proteins (WW34, Ref2NM, MAGOH, ORF57, Y14, and TAP) over a range of conditions and found that the equimolar addition of the charged *L*-amino acids Arg and Glu increased protein solubility and long-term stability without altering the ability to interact its target proteins or RNA. Furthermore, the simultaneous addition of these two amino acids to the buffer reduced aggregation of these proteins during concentration processes and protein degradation, resulting in increased formulation stability [7]. These findings were reported for all the proteins in the study, including WW34. Heteronuclear single quantum correlation (HSQC) NMR studies showed that the addition of Arg and Glu to buffer did not cause any structural alteration of the proteins over a period of several weeks.

The synergistic effects for the combined use of arginine and glutamic acid to increase protein solubility was further described by Shukla *et al.* [8] using preferential interaction coefficients (Γ_23_) and molecular dynamics. It was thought that interactions between the two amino acids (Arg and Glu) increase their presence on the protein surface, therefore reducing the number of protein–protein interactions. The synergistic effect of two amino acids used as excipients increased the number of Glu and Arg molecules close to the protein surface when compared to the individual addition of Arg or Glu only, and this synergistic effect was reported to be responsible for an increased solubility effect on WW34 [8]. More relevant to our work, the location of excipient molecules close to the nearest amino acid on the protein surface was determined using molecular dynamics simulation [9], and those were reported as excipient binding sites. These sites were taken as the benchmark for our molecular docking methodology.

We report the development of a computational approach for excipient selection by identifying hotspots for their interaction on the protein surface. The hotspots, preferential binding surfaces for excipients, were identified using molecular docking. The use of this approach is further exemplified by prediction of Fab hotspots for commercially available excipients. The excipients were used as ligands and were selected from those used in commercial formulations of therapeutic proteins [9].

## 2. Results and Discussion

Protein aggregation often involves non-covalent protein–protein interactions early in the process where nucleation, or the establishment of interacting multiple protein species, occurs. The process of aggregation occurs more readily in unfolded forms of a protein where there are more hydrophobic interactions possible than in the folded, active form of the protein. The tertiary structure of a folded protein is dynamic because it is reliant on non-covalent intramolecular interactions, so specific aggregation pathways are protein-dependent. Generally, the specific steps resulting in protein aggregation are poorly understood [10,11], but it has been shown that low molecular weight substances can delay or decrease aggregation [12] and protein unfolding [13]. The protective effect can be a result of new intermolecular interactions formed between additives, such as excipients, and the protein [14]. It is well established that the formation of intermolecular interactions and their location on the protein surface can be studied *in silico* using molecular docking [15].

Molecular docking is a methodology, which allows the study of the interactions between different molecules with many software packages available for this type of study. Knowledge of the molecular system is crucial for the decision about what type of software package to use. In general, it is possible to classify the software packages based on the molecular system that they are generally applied to. Often they can be either directed towards protein–protein (protein–DNA) interactions or small molecule–protein interactions. All docking software can perform either (i) rigid docking, where the conformation of the molecules is not altered during the docking process; or (ii) flexible docking, where the conformation of part or whole molecules change during the best-fit search.

There are two stages in a molecular docking protocol: sampling and scoring [16]. During sampling, a range of different conformations of a ligand (flexible docking only) and their poses, orientations of a ligand in respect to the binding surface of the target, are generated. In the second step, these poses are scored and ranked according to a mathematical algorithm used to predict the binding affinities. The last step is where most software packages diverge and where the most research has been invested [17,18,19]. Docking software packages are usually evaluated based on the accuracy with which they predict poses and the calculation of free energy between target and ligand.

Scoring functions of both protein–ligand and protein–protein docking software can be classified according to how the energy terms are derived [20]. These can be force-field-based, empirical, or knowledge-based. Force-field-based scoring functions determine physical atomic interactions in the system, such as Van der Waals, bond terms (length, angles and torsions), and electrostatics derived from force-field parameters. Force fields allow the determination of the energy of a system by calculating all the forces influencing the system using classical physics and sometimes higher-level quantum mechanics, and experimental data [18,21]. Empirical scoring functions make use of weighted energy terms to determine binding affinities [18,22]. These functions deconvolute the free energy between receptor and ligand (Δ*G*) into a sum of the weighted (ω_i_) free binding energies for each component (e.g., electrostatics, hydrogen bond) that contribute to the interaction (Δ*G*_i_) [23]. The weighting coefficients are derived by fitting the predicted binding affinity data with the experimental data (crystal structures of complexes) using multivariate regression analysis [18,23]. Finally, knowledge-based scoring functions derive the energy function from known structures of complexes (protein–protein, protein–ligand). Statistical analyses are then applied to derive pairwise potentials obtained directly from experimental data [18]. The energy potentials for the pairwise atomic interactions are derived as a function of the distance between the atoms and are then used to calculate the energies of all conformations [24].

To demonstrate the potential of using docking to gain a better understanding of interactions involved in protein formulations, we applied protein–protein and protein–ligand docking on the WW34 protein with known interactions with two amino acids—Arg and Glu.

Shukla *et al.* [8] combined available experimental information [7] with their molecular dynamics simulations to evaluate the sites of interaction and excipient ratio, providing a approach that addressed the issue from a different aspect. They also employed the preferential interaction theory to assess the effect of excipients on protein solubility. The correlation between preferential interactions between protein and excipients and protein solubility has been previously studied by Arakawa *et al.* [25]. Briefly, they showed that, if the protein is preferentially hydrated (Γ_23_ < 0), the interaction with excipients is less favoured and the interaction between protein molecules is favoured to prevent excipient interactions with the protein surface. Increased protein–protein interaction and decreased protein–excipient interaction can often result in a decrease of protein solubility. Shukla *et al.* [8] performed molecular dynamics simulations of the WW34 protein in the presence of varying molar concentrations of Arg and Glu and equimolar ratios of the two excipients. The theoretical preferential interaction coefficient for each system was determined from the number of excipient molecules and water molecules as a function of their distance from the protein surface throughout the trajectory [8,26]. The authors considered the relationship between the preferential interaction coefficient and protein solubility previously defined by Shulgin *et al.* [27]. The preferential interaction coefficient determined for the protein in an equimolar mixture of L-Arg (Γ_23_ = 8.8) and L-Glu (Γ_23_ = 3.6) was higher than for systems with only one of the excipients (Arg, Γ_23_ = 1.3–7 and Glu, Γ_23_ = 0.5–0.9). This was due to the interactions between Arg and Glu, thus supporting that the synergistic effect of Arg and Glu enhanced the local concentration of the amino acids on the protein surface, leading to an increased solubility of WW34 [8]. The number of excipient molecules close to the protein surface was calculated by determining the nearest amino acid to each one of the excipient molecules in the simulation. The authors found that there were few sites where both Arg and Glu were present, allowing them to establish the binding of the motifs. These interaction sites were the amino acids GLU 40, ASP41, ARG79, and ARG71 [8]. The identified sites of interactions have been used as a benchmark to evaluate the effectiveness of our approach.

In this study, the interaction surfaces were determined using the molecular docking, and four different software packages were utilised for validation purposes. Two types of molecular docking were employed: the rigid docking to determine protein–protein interactions and flexible docking to evaluate protein–excipient interactions. For each category, two distinct software packages were used that generate ligand poses (excipient–protein docking) or different types of scoring functions (protein–protein docking) and evaluate the consistency of the observed poses. This provides complementary information that should ensure the identification of possible relevant interaction sites and aid the rational selection of excipients and the design of pharmaceutical formulations. The details of the scoring functions for each one of the software packages included in the study can be found in Section 3.

### 2.1. Hotspots for Protein Interactions with Excipients—Flexible Docking

Most protein-small molecule docking software requires some information about the binding site for accurate results. Even though some packages allow for expansion of the area in the protein to be considered, usually by expanding a grid or box, this leads to a loss of definition. The larger the area, the less points within the box will be considered, thus potentially compromising the results. Software packages where the whole protein must be screened for potential sites of interaction with excipients would not suit the aim of this study. To develop the docking method, two different protein-small molecules software packages were initially used: GLUE [28] and iGemDock [29].

The two software packages selected both allow whole protein docking without requiring information on the binding site. This is crucial in this approach to rationalise screenings of excipients of different classes that might be needed to stabilise a given protein. This means that there will likely be more than one hotspot (binding site) for different classes of excipients. For this study, the interactions of arginine with the target protein were evaluated and compared with previously published data [8].

**Figure 1:**
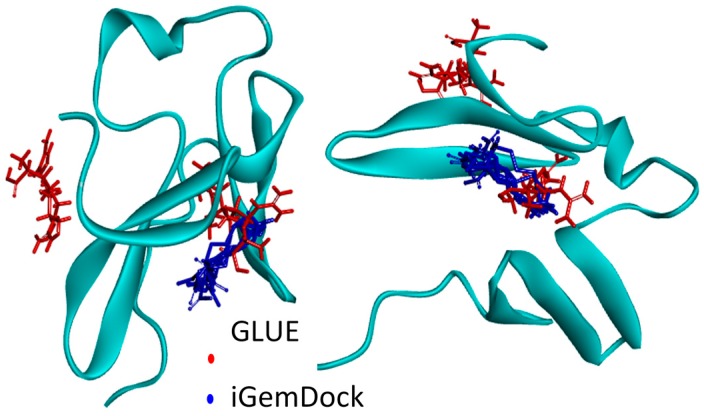
Frontal and side view of the molecular docking solutions of WW34 (green) interactions with arginine obtained with GLUE (**red**) and iGemDock (**blue**).

**Figure 2:**
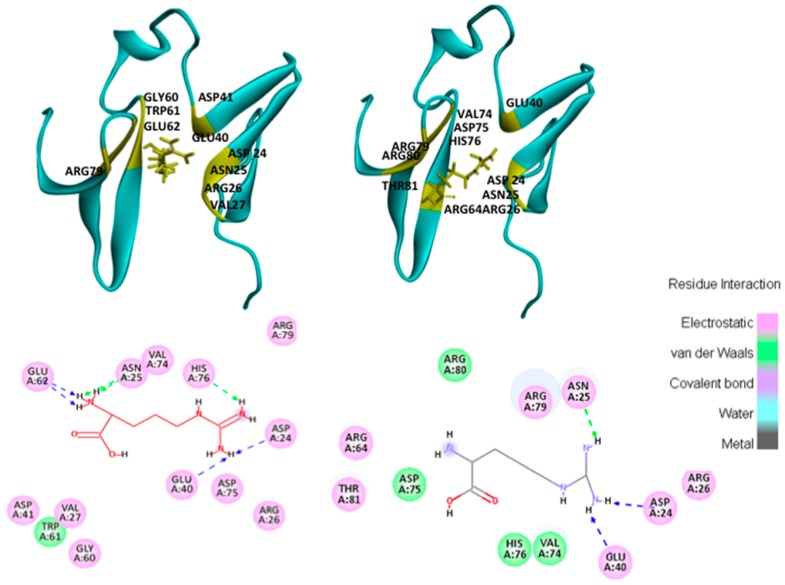
Lowest energy solutions of WW34 interactions with arginine (yellow tube representation). In yellow residues, less then 4 Å was found from the structure set as receptor (green ribbons). Results obtain with GLUE (**left**) and iGemDock (**right**). The diagrams in the bottom represent the interactions network between the arginine molecule and the surrounding residues from WW34, for the solutions above. The different colours correspond to the nature of the interaction as described in the legend.

The results obtained with the GLUE software package identified two preferential sites for arginine interaction, while iGemDock solutions were all in the same site (Figure 1). Nonetheless, the lowest energy solution for both software packages was found to be in the same site (Figure 1). A detailed analysis of intermolecular interactions for this hotspot revealed that the orientation of the arginine in docking poses obtained for two docking software is different (Figure 2).

The residues that interact with arginine are the same as previously reported [7,8]. The orientation of the arginine molecule appears to be different. This may be due the scoring functions, which account for the electrostatic contributions differently. Nonetheless, these preferential interaction sites on WW34 can be considered to be hotspots for arginine.

Previous studies by Shuka *et al.* showed that arginine molecules can form hydrogen bonds with residues ASP41 and GLU40 [8]. The interaction map obtained with the visualiser showed the potential for hydrogen bond formation between arginine and GLU40 in both GLUE and iGemDock. However, only GLUE found ASP41 at a distance, which would suggest electrostatic interaction between arginine and this residue. It is necessary to bear in mind that these are static points, and, even though the distance between arginine and ASP41 was only identified for this kind of interaction, in a real world situation, structures are dynamic and distances can change, leading to closer contacts. Further molecular dynamics studies with solutions from GLUE were performed to investigate the validity of the results obtained.

### 2.2. Hotspots for Protein–Protein Interactions—Rigid Docking

Two molecules of WW34 were docked against each other to identify the potential regions and residues that could be involved in interactions between two protein molecules and could be responsible for protein aggregation. This would allow us to evaluate if a protein–protein interface is the same as the arginine interaction site on the protein surface identified with protein–ligand docking (Section 2.1). Therefore, this would provide a good case study to demonstrate how the presence of this excipient is effective in preventing such protein–protein interactions and stabilising WW34.

A number of different computational approaches have been developed to identify protein regions responsible for aggregation. However, most of these methods utilise available experimental data and bioinformatics tools to identify sequences of aggregation-prone regions (APR) and do not take protein tertiary structure into account [30]. Molecular dynamics and Monte Carlo simulations have also been used for aggregation propensity studies [30,31]. However, these are time-consuming approaches that cannot be easily translated in the early stage formulation development environment and hence would not be efficient for excipient selection.

We chose to utilise rigid protein–protein docking software, as it is fast and based on Fourier transform algorithms, minimising the computational time required for the identification of surfaces that could be responsible for aggregation. For validation purposes, two independent rigid docking software packages were used: Hex and GRAMMX. The main difference between these is how the surfaces of the receptor and ligand are defined and the approximations used for electrostatic contribution.

**Figure 3:**
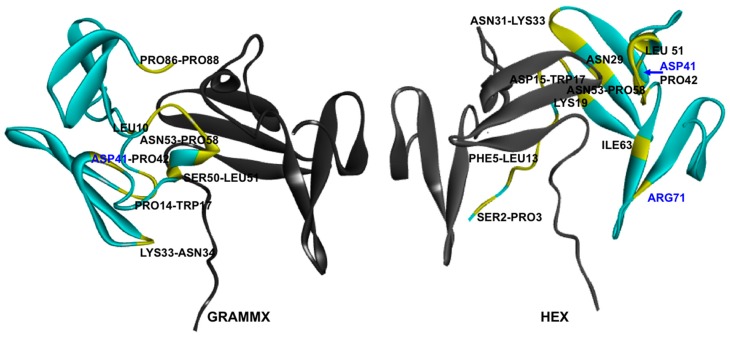
Molecular docking results of two WW34 molecules (green set as ligand and grey set as target) depicting protein–protein interactions that could be responsible for aggregation. The residues found to be less than 4 Å from the structure set as receptor are colored in yellow. GRAMMX, lowest energy solution (**left**); HEX, lowest energy solution (**right**).

These two software packages use different scoring functions to rank the results found; therefore, it would be expected that the orientation of the molecules given by HEX and GRAMMX would be different, which can be observed in the lowest energy solutions for the two different packages (Figure 3). However, despite these differences in the orientation of the two WW34 subunits under study, the key areas for the interactions that may be responsible for aggregations are similar (Figure 3). A closer look into residues less then 4 Å apart for the lowest energy solutions found by HEX and GRAMMX showed that ASP41 and PRO42 were involved in interactions between subunits. The same ASP41 has been found to be involved in interactions with arginine (Arg) and glutamic acid (Glu) in the published molecular dynamics study by Shukla *et al.* [8]. The authors showed that Arg and Glu interacted with the protein surface, reducing the number of protein–protein interactions. Amongst the identified arginine and glutamic acid binding motifs was residue ASP41, which was also identified by our methodology, giving a good indication of the suitability of the method.

### 2.3. Molecular Dynamics Simulations—Validation of Identified Hotspots

Molecular docking software packages are designed to find and rank interactions between two molecules. This means that we will always obtain solutions. The energy values obtained are crucial to judge the reliability of the solutions obtained. For complexes of large molecules (e.g., protein–protein) or for detailed analysis of defined and known binding sites, differences in energy values can discriminate the docking interactions. However, when docking small molecules, the differences in energy values between solutions or even in its absolute value can be low and therefore not enough to confidently judge on an interaction. Molecular dynamics simulations can aid the understanding of protein–excipient interactions for systems that are well defined, as demonstrated with 50-ns simulations of bovine serum albumin in the presence of two excipients [32].

**Figure 4:**
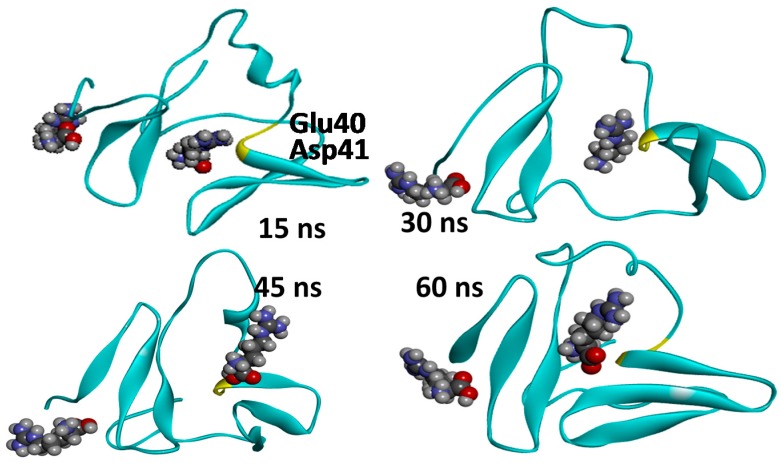
Snapshots of the 60-ns MD simulation performed with the solutions obtained from GLUE. Two Arg molecules were considered so that both hotspots identified by GLUE were represented. WW34 protein is displayed as ribbons in green and the two arginine molecules are displayed as CPK and coloured by element. The reference residues Glu40 and Asp41 are highlighted in yellow in each snapshot.

To evaluate the dynamic behaviour of the identified interactions and to verify that the excipient will preserve interactions observed in binding poses identified by the docking protocol, further validation of the hotspots was conducted. The molecular dynamics simulation was performed using one of the solutions obtained from GLUE, WW24, and two arginine molecules as a starting point. The MD simulation was carried out on a fully solvated system using explicit solvent at 300 K and 1.03 bar under constant number of particles, pressure, and temperature (NPT) conditions. Although we did not evaluate the binding energy during the simulations, this allowed us to observe the interaction between arginine and the protein residues over the period of the simulation. From the trajectory analysis, it was possible to observe that whilst both WW34 and arginine change conformation, the interaction with the key identified residues, GLU40 and ASP41, is maintained throughout the simulation (Figure 4), namely the distance between selected residues and arginine always being lower than the 4-Å cut off.

### 2.4. Application to the Fab A33 and a Set of Commercial Excipients

The method was then applied in an effort to determine the hotspots for interactions between Fab A33 [33] and several excipients. Fabs are an emerging class of antibody fragments developed for therapeutic applications. There are already some examples in the market such as Cimzia (UCB) and Lucentis (Genentech), amongst others, and more are being developed [34]. Fabs can be expressed in microbial systems, making their production process more economical in comparison to monoclonal antibodies (mAbs) [34]. Other advantages for their use include faster tissue penetration [35,36] and the absence of the Fc portion of antibodies [34]. The use of Fabs as therapeutics is growing, making them an interesting case study for the application of the computational method developed. Furthermore, they are easier to model as complex glycosylation patterns, and flexible domains found in mAbs are not present.

The excipients selected have all been approved by FDA for parental use. As a starting point, we utilised those that are commonly used in commercial formulations. Most of these excipients have a stabilising role in protein formulations. Representative excipients from different classes were chosen: two amino acids, two saccharides, two sugar alcohol isomers, and two surfactants. For the docking experiments, the following excipients were used as ligands: arginine; glycine; sucrose; trehalose; manitol; sorbitol; polysorbate 20; polysorbate 80.

GLUE software was used to establish the preferential spots of interaction between ligands and A33. The ligands and there interaction with A33 were firstly compared and analysed based on their structure and/or function similarity.

#### 2.4.1. Hotspots for Fab A33 Interactions with Two Amino Acids

Arginine is frequently used in parentally administered formulations as a viscosity reducer [13]. However, arginine is also a mild detergent that can bind to proteins and have a negative effect, namely in protein thermo stability [13,37]. Glycine (Gly) is routinely used in lyophilised protein formulations, as it improves porosity and coherence of the cake [38,39,40].

**Figure 5:**
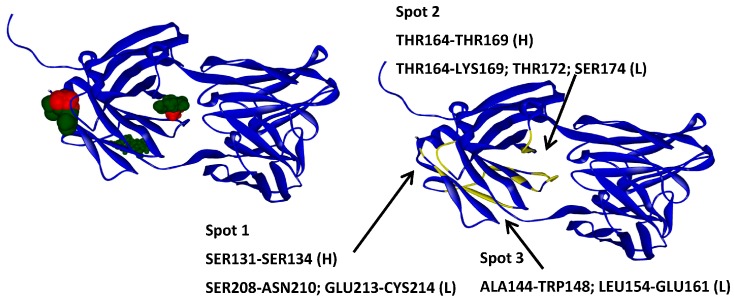
Molecular docking results for arginine and glycine obtained with GLUE software. A33 is displayed in blue ribbons, arginine in green and glycine in red (**left**); The higher affinity molecules are displayed as larger CPK. A33 residues involved in each identified spot (**right**).

Arginine was found to interact in three different spots in the protein and five different solutions were retrieved (Figure 5). On the other hand, glycine retrieved only three solutions but with higher affinity (Table 1) and only two spots (Figure 5), indicating higher specificity than arginine.

#### 2.4.2. Hotspots for Fab A33 Interactions with Two Saccharides

**Figure 6:**
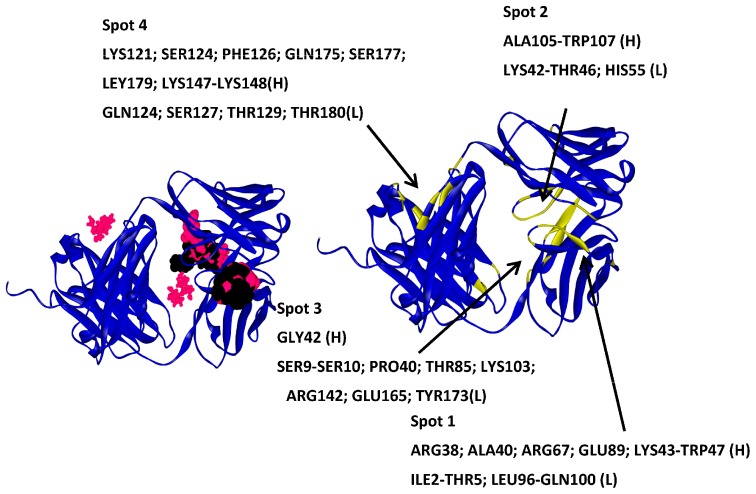
Molecular docking results for trehalose and sucrose obtained with GLUE software. A33 is displayed in blue ribbons, trehalose in black, and sucrose in pink (**left**); The higher affinity molecules are displayed as larger CPK. A33 residues involved in each identified spot (**right**).

Trehalose and sucrose are non-reducing disaccharides that are commonly used for the freeze-drying of therapeutic proteins for parenteral administration, namely monoclonal antibodies [41,42,43,44]. A total of 15 solutions were obtained for trehalose and 13 for sucrose. Whilst for trehalose the lowest energy solutions all interacted on the same two spots, for sucrose four spots were found for the five lowest energy structures, showing much lower specificity in the interaction (Figure 6; Table 2).

#### 2.4.3. Hotspots for Fab A33 Interactions with Two Sugar Alcohol Isomers

**Figure 7:**
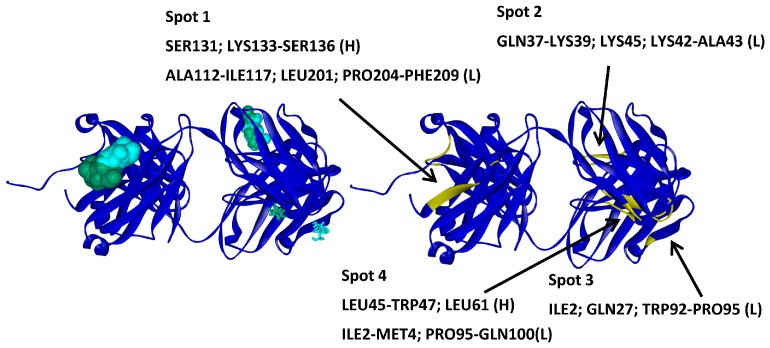
Molecular docking results for mannitol and sorbitol obtained with GLUE software. A33 is displayed in blue ribbons, mannitol in green, and sorbitol in light blue (**left**); The higher affinity molecules are displayed as CPK and lowest as tubes. A33 residues involved in each identified spot (**right**).

Mannitol and sorbitol are monosaccharide-derived polyols that are commonly used as pharmaceutical excipients (e.g., diluents in tablets). More relevant to this study, these are also used as carriers to improve cake homogeneity and the appearance of lyophilised products [45,46,47]. Despite being isomers, slight differences are often observed between mannitol and sorbitol. Mannitol displayed a higher number of solutions (19) than sorbitol (18), and an overall higher affinity (Table 3). Both molecules mostly interacted in the same two hotspots for the top five docking solutions, except from the structures with lower binding affinity, which interacted in a different spot (Figure 7).

#### 2.4.4. Hotspots for Fab A33 Interactions with Two Surfactants

Polysorbates are effective at preventing physical and chemical instability during processing and freeze-thaw cycles, even at very low concentrations, and they have relatively low toxicity [48].

**Figure 8:**
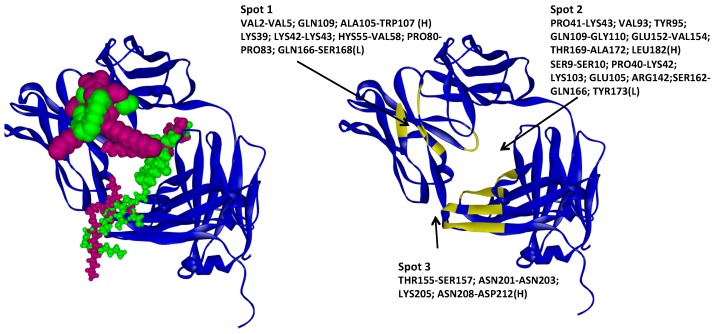
Molecular Docking results for polysorbate 20 and 80 obtained with GLUE software. A33 is displayed in blue ribbons, polysorbates are represented in green and purple (**left**); The higher affinity molecules are displayed as CPK and lowest as tube or ball and stick. A33 residues involved in each identified spot (**right**).

There were no differences between polysorbate 20 and 80 in term of number of solutions, interaction spots or binding affinity (Figure 8; Table 4).

### 2.5. Hotspots for Fab A33 Interactions with Excipients

**Figure 9:**
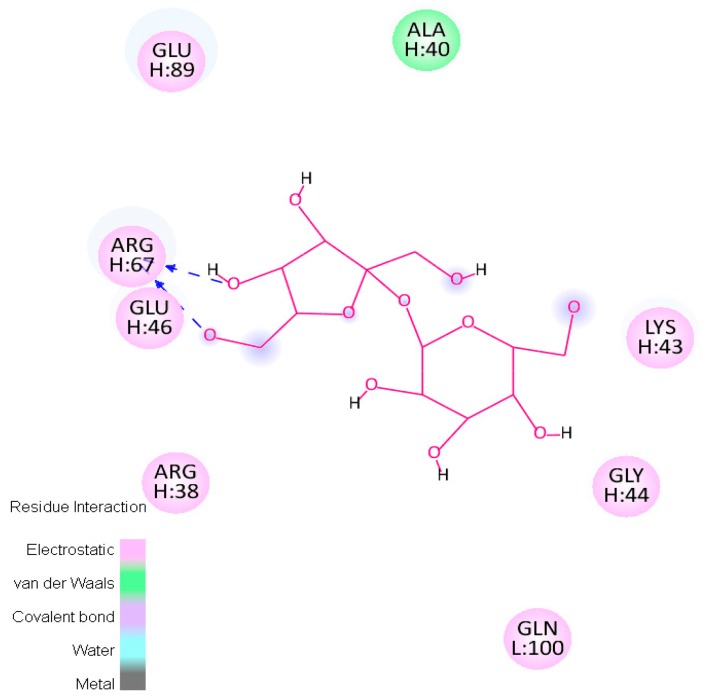
Example of the interaction framework plotted—in this case, sorbitol.

In addition to the above analysis where the number of docking solutions and spots of interactions were analysed for each excipient in the study, the three highest affinity solutions for each ligand were further inspected. The interaction framework of these molecules and the interaction spot on A33 was determined and plotted. This allowed for the determination of the A33 residues involved to evaluate the nature of the interactions in an effort to understand the differences observed in the binding energy values for each excipient. Figure 9 shows an example of this interaction framework. In this example, it is possible to see how the relatively high-energy values observed for sorbitol correspond to a low binding affinity and reflect the weak nature of its interactions with the protein.

**Figure 10:**
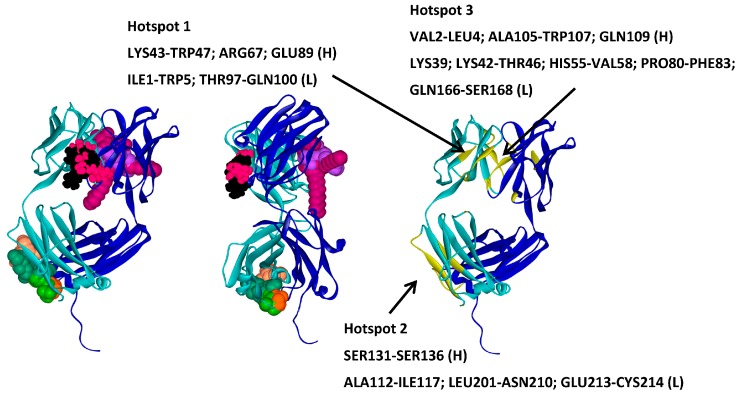
Frontal (**left**) and side (**center**) views of the three hotspots for A33–excipient interaction identified. A33 residues involved in each hotspot highlighted in yellow (**right**). A33 is represented as ribbons with the heavy chain in dark blue and the light chain in green. The representative structures of the excipients are displayed in CPK, trehalose—black; sucrose—pink; arginine—light green; glycine—orange; manitol—salmon; sorbitol—dark green; polysorbate 20—magenta and polysorbate 80—purple.

With all the information collected, it was possible to identify and define three hotspots for the interaction of A33 with the excipients (Figure 10; Table 5): one hotspot where sucrose and trehalose interacted most favourably, a second hotspot where both saccharides and amino acids interact, and finally the third hotspot where only surfactants docked (Figure 10). Hotspots 2 and 3 are located mainly in the light chain of A33 (Table 5).

### 2.6. Protein–Protein Interaction Surfaces between Two Fab A33 Molecules

**Figure 11:**
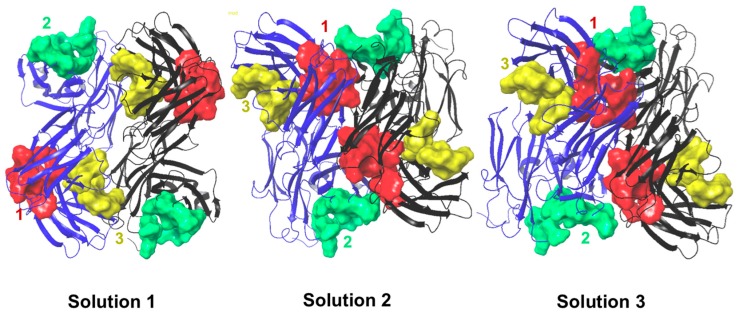
Docking solutions for interaction between two A33 molecules obtained with Hex. The A33 molecule defined as target is displayed in blue ribbons, and the molecule defined as ligand in grey. The hotspot surfaces are shown for both molecules and coloured according to the following scheme (Hotspot 1—**red**; Hotspot 2—**green**; and Hotspot 3—**yellow**).

Hex was used to perform protein–protein docking with Fab A33. This allowed further validation of the hotspots by evaluating if the residues that were involved in the interaction between two Fab A33 molecules were the same as the ones interacting with the excipients. Figure 11 shows one of the obtained solutions from the molecular docking experiment. For this analysis, the three lowest energy solutions were considered (Table 6).

The hotspots found for the protein–excipient interactions are located in regions prone to interactions between two A33 molecules. These interactions potentially lead to protein aggregation that can be prevented if the excipients are added to the protein.

### 2.7. Experimental Confirmation of the Presence of Interaction between A33 and the Commercial Excipients

Thermal analysis was conducted with the Fab A33 in the presence and absence of excipients to quickly and qualitatively assess whether there was an effect of the presence of the excipients on the Fab melting temperature (*T*_m_) to further support the docking results. Protein *T*_m_ can be affected by a variety of factors and can be used to monitor unfolding and aggregation phenomena [49]. Excipients are known to impact the thermodynamic behaviour of proteins and affect *T*_m_ values [13]. The melting temperature of A33 and combinations of A33 and the excipients used in the docking calculations was determined using the OPTIM 1000 (Avacta). These experiments aimed to demonstrate only the presence of an effect driven by protein–excipient interactions, since no screening for the optimisation of the stoichiometry between protein and excipient was conducted.

Table 7 shows the *T*_m_ values determined for each of the conditions tested. Overall, all excipients, except Arg, appeared to positively affect the *T*_m_ of A33. The adverse effect of Arg in A33 *T*_m_ value was expected since, as mentioned above, arginine can act as a mild detergent [13,37].

**Figure 12:**
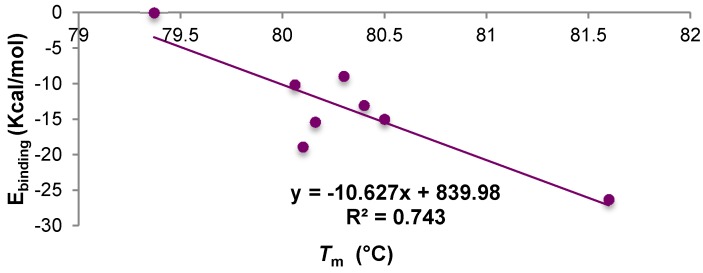
Correlation between molecular docking results, expressed as a binding affinity and experimental results, *T*_m_.

Without screening the concentration of the excipients and the proportion with the protein, it is not possible to make quantitative considerations on this effect or which excipient can provide better results. However, these results further confirm an interaction between excipients and protein and their potential to stabilise protein. Finally, the docking results for all excipients except arginine (negative control, different mechanism) were correlated with the melting temperatures (Figure 12). The correlation shows that the computational method described was able to describe 74% of the experimental space, providing a basis for the rational selection of excipients in formulation design experiments.

## 3. Materials and Methods

### 3.1. Molecular Structures Setup

The structures of all excipients were drawn in Maestro 10.3 (Schrodinger, LLC, New York, NY, USA) and a short minimisation protocol was performed using Macromodel 10.0 (Schrodinger, LLC, New York, NY, USA). The *Drosophila* Su(dx) (WW34) structure was taken from PDB 1TK7 [50]. The 3D structure of Fab A33 is a homology model previously built at UCL. The protein structures were verified and optimised with the Prep Wiz tool from Maestro 10.3 (Schrodinger, LLC, New York, NY, USA).

### 3.2. Protein-Protein Molecular Docking

The docking of two protein molecules to determine interaction surfaces that could be responsible for the aggregation was performed using Hex; with its shape and electrostatic protocol, the scan steps were set to 0.75 with two substeps. The docking correlation order was set to 25 for steric scan and 25 for the fine search. The grid size was set to 1, and 100 solutions were recorded. Of these, the first structures of the first 10 clusters were saved as PDB files. The same experiment was performed using the web server GRAMM-X (Lawrence, MA, USA). The results were processed using Discovery Studio 4.0 (Biovia, San Diego, CA, USA). A 4-Å cutoff was allowed to identify residues involved in protein–protein interactions.

The Hex software package was used for the protein and nucleic acids interaction studies, though it also allows small protein–ligand interactions with a rigid ligand. Hex is a fast Fourier transform (FFT) docking correlation-based program; however, unlike previously developed programs, it uses soft polar Fourier correlations, minimising the computational time required to explore the Cartesian space [51,52]. The molecular surface of the proteins are represented by an internal and an external “skin”, each one of them being represented by a Fourier series comprising radial and spherical harmonic basis functions [51]. Electrostatic contribution is optional, but, when present, it is only taken into account in the final search, having a small weight in the final scoring function. The resulting structures are ordered from lowest to highest energy, and the structures are then clustered with a 3-Å threshold for the main chain C_α_–C_α_ RMSD values [53].

GRAMM-X is also a fast Fourier transform (FFT)-based software that applies smoothed Lennard Jones potentials, refinement, and knowledge-based scoring. With this package, proteins are represented by a fine-grid softened by Lennard-Jones potential function calculated for a probe atom:

(1) $$V_{ij}\left(r\right)=\frac{1}{{\alpha \sigma }_{ij}^{6}+r^{6}}\;\left(\frac{4\varepsilon_{ij}\sigma_{ij}^{12}}{{\alpha \sigma }_{ij}^{6}+r^{6}}-4\varepsilon_{ij}\sigma_{ij}^{6}\right)$$

The optimal values of the parameters for a typical protein in unbound conformation are α = 0.4, σ = 0.33 nm, and ε = 0.5, these are applied to all non-hydrogen atoms. The top 4000 grid-based predictions are subjected to a conjugate gradient minimisation in continuous 6D rigid body space with the same soft potential [54]. The minimisation accumulates many points—some in local minima. One representative prediction for each minimum is stored, and the number of initial predictions falling into this minimum is marked as the volume of the minimum. The average radius of such minima on the smoothed landscape is 5 Å. For each minimised prediction, the following terms are calculated: the soft Lennard-Jones potential, the evolutionary conservation of predicted interface, the statistical residue–residue preference, the volume of the minimum, the empirical binding free energy, and the atomic contact energy [54]. To eliminate predictions that are likely to be located far from the correct binding site, a support vector machine filter is applied. The remaining predictions are then re-scored by a weighted sum of the potential terms [54].

### 3.3. Protein–Excipient Molecular Docking

Molecular docking with GLUE was performed using GRID protocol. The three components of the GRID (Molecular Discovery Ltd., London, UK) protocol were used in these studies. GREATER (Molecular Discovery Ltd., London, UK) was used to generate the KOUT files for all molecules involved. GLUE (Molecular Discovery Ltd., London, UK) requires either KOUT or MOL2 files for the docking process. For this study, the PDB files were converted to KOUT files. For the docking process, with GLUE, all available probes were selected, and a maximum number of 100 binding sites were used with an energy cut off of −100 Kcal/mol. The maximum iteration value was set to 120. For ligand flexibility, 5 rotatable bonds were allowed, and electrostatics was included. Arginine and the selected commercially used excipients were used as ligands. The *Drosophila* Su(dx) structure (1TK7) and homology model of A33 were set targets. The same structure was docked with iGemDock (BioXGem, Hsinchu, Taiwan). The docking accuracy was set to the “Accurate Docking” setting by choosing a population size of 800 with 10 generations to produce 10 solutions. The docked ligand conformations were then re-ranked for the post-docking analysis. A 4-Å cutoff was allowed to identify residues involved in protein–protein interactions. A cutoff of 3.5 Å was used to build the interaction maps involved in the interaction with excipients.

GRID is a calculation-based procedure that allows for the determination of energetically favourable binding sites on a molecule with a known structure. GRID has been used to study nucleic acids and individual molecules such as drugs or macromolecules (such as proteins) [28]. GRID also enables the study of several different molecules consecutively. GRID results can be visualised with GView, which allows molecular interaction fields, GRID energy contributions due to atoms of the target, and molecular structures with distances, torsion, and dihedral angles to be viewed. The graphical user interface (GUI) provides access to the GRID software package and is strongly integrated with GView, helping users to visualise the structure of the Target and the results of the GRID computation at the same time. GLUE is a GRID-docking programme capable of finding possible interaction sites between a molecule set as “target” and a small molecule called a ligand. It requires the input of the 3D-structures of both target and ligand, and it allows visualisation of the docking process. Its scoring function takes into account steric repulsion energy, electrostatics contribution, a dry parameter, which accounts for hydrophobic energy, and an additional hydrogen bonding charge-reinforcing parameter [28].

The second docking package that was evaluated is iGemDock [29]. This flexible ligand-docking software uses an empirical scoring function and a genetic evolutionary method for molecular docking [29,55]. The energy function can be described by

(2) $$E_{\text{tot}}=\;E_{\text{bind}}+\;E_{\text{pharma}}+\;E_{\text{ligpre}}$$

where *E*_bind_ accounts for inter- and intra-molecular energies and an additional *E*_penal_, and, in case the ligand is outside the search box, its fixed value is 10,000. The *E*_pharma_ term is the sum of all hot-spot atoms of the interaction between ligand and protein:

(3) $$E_{\text{pharma}}=\;\overset{lig}{\underset{i-1}{\sum }}\overset{hs}{\underset{j-1}{\sum }}f\left(w_{j},\;B_{ij}\right)F\left(r_{ij}^{B_{ij}}\right)$$

where *w_j_* is the pharmacophore weight of the hot-spot atom *j*, $r_{ij}^{B_{ij}}$ is the distance between the atoms *i* and *j* with the interaction type *B_ij_* forming by the pair-wise heavy atoms between ligands and proteins; *B_ij_* is either a hydrogen bond or a steric state; *lig* is the number of heavy atoms in the ligand; and *hs* is the number of hot-spot atoms in the receptor. The value of *f*(*w_j_, B_ij_*) is *w_j_* or 0. *f*(*w_j_, B_ij_*) is *w_j_* if the interaction type (*B_ij_*) equals the type of hotspots found on the target receptor. The ligand preferences include electrostatic (*i.e.*, the number of electrostatic atoms) and hydrophilic characteristic (*i.e.*, the atom numbers of hydrogen donor and acceptor). The *E*_ligpre_ is a penalty value for a ligand, which is unable to satisfy the ligand preferences and is defined as

*E*_ligpre_* = WP*_elec_* + WP*_hb_ (4)

where *WP*_elec_ and *WP*_hb_ are the penalties for the electrostatic and hydrophilic preferences, respectively [56]. iGemDock required the input of the coordinates of target protein atoms from the PDB format file and sequentially read the atom coordinates of a ligand from the prepared ligand database. The ligand database and the target protein were prepared and sequentially performed the flexible docking for each ligand by using the genetic algorithm implemented in GemDock [30]. Its energy function consists of electrostatic, steric, and hydrogen-bonding potentials. The latter two terms use a linear model that is simple and recognises potential complexes rapidly [29].

### 3.4. Molecular Dynamic Simulations

Molecular dynamics simulations were performed with the top scores for 1TK7 and arginine from both GLUE and iGemDock. The simulations were performed with Desmond [57] within the Maestro environment. The molecular dynamics simulation, minimisation, and relaxation steps were performed for 60 ns at 300 K and 1.03 bar, and in a buffer zone of 10 Å. Snapshot structures were recorded every 5 ps. The simulation event analysis tool for trajectory analysis within Maestro (GUI) was used to perform RMSD and RMSF calculations and measurements such as hydrogen bonds, distances, and gyration radius along the trajectory. Snapshots from the trajectory were exported every 15 ns for further analysis with Discovery Studio (Accelerys). A 4-Å cutoff was allowed to identify residues involved in protein–excipient interactions.

Molecular dynamics methods calculate the forces between the atoms at each time-step, and from that these methods are designed to calculate the new positions and velocities of all of the atoms by applying classical laws of physics [58]. Desmond is a MD code, which allows parallel scalability for clusters, improving simulation throughput, and reducing computational time. It comprises a series of methods and algorithms that accelerate MD simulations, including a parallel decomposition method and message-passing techniques that reduce communication requirements, as well as novel communication primitives that further reduce communication time. Its numerical techniques maintain high accuracy while using single precision computation in order to exploit processor-level vector instructions.

### 3.5. Stepped Thermal Experiments to Determine T_m_ Values

Chemicals including trehalose, sucrose, glycine, arginine, mannitol, sorbitol, Tween 20, Tween 80, and sodium phosphate were purchased from Sigma-Aldrich (Poole, Dorset, UK). Excipients, sodium phosphate and purified Fab were prepared in stock solutions and mixed together to reach a final concentration of 1 mg/mL of Fab in 10 mM phosphate buffer at pH 7. The final concentration of excipient was variable: 5% (*w*/*v*) for trehalose and sucrose, 2% (*w*/*v*) for glycine and arginine, 4% (*w*/*v*) for mannitol and sorbitol, and 0.4% (*w*/*v*) for Tween 20 and Tween 80. Avacta OPTIM 1000 (Thorp Arch Estate, Wetherby, UK) was used to perform the stepped thermal unfolding study. The samples were ramped from 20 to 90 °C at 1 °C per step, with a temperature tolerance of 0.2 °C. *T*_m_ was determined as described by Chakroun *et al.* [59].

## 4. Conclusions

A combination of molecular docking methods has been successfully utilised to predict hotspots in protein excipient interfaces in a *Drosophila Su*(*dx*) protein (WW34). The positive effect of the presence of excipients in the proximity of these hotspots was confirmed by molecular dynamics simulations, thereby validating this approach for predicting interaction interfaces. Furthermore, possible favourable interactions between Fab A33 and several excipients predicted by docking were confirmed qualitatively by *T*_m_ values from a stepped thermal study. The computational methods that provide information about the intermolecular interactions between excipients and proteins could play an important role in the design of therapeutic protein formulations during development. Molecular docking provides the means for the rapid identification of molecules that can form favourable interactions with protein surfaces, hence enabling a rational approach in the design of experimental formulation development and a more effective use of the small amounts of protein therapeutics that are usually available in early stage development.

## Figures and Tables

**Table 1:** Binding affinity and hotspot for each arginine and glycine solutions.

Molecule	Arg1	Arg2	Arg3	Arg4	Arg5	Gly1	Gly2	Gly3
*E*_binding_ kcal·mol^−1^	−25.029	−22.737	−19.848	−15.913	−15.583	−27.304	−26.352	−25.334
Spot	3	1	3	2	2	1	2	1

**Table 2:** Binding affinity and hotspots for the five lowest energy solutions found for the trehalose and sucrose solutions.

Molecule	Tre1	Tre2	Tre3	Tre4	Tre5	Suc1	Suc2	Suc3	Suc4	Suc5
***E*_binding_** kcal·mol^−1^	−16.638	−13.783	−13.023	−12.262	−11.762	−20.233	−16.780	−16.334	−13.868	−13.286
Spot	2	1	2	1	1	2	1	1	3	4

**Table 3:** Binding affinity and hotspots for the five lowest energy solutions found for mannitol and sorbitol solutions.

Molecule	Man1	Man2	Man3	Man4	Man5	Sor1	Sor2	Sor3	Sor4	Sor5
*E*_binding_ kcal·mol^−1^	−11.901	−10.67	−8.537	−8.415	−7.852	−9.879	−9.372	−8.904	−8.524	−8.329
Spot	1	1	2	3	1	2	1	1	1	4

**Table 4:** Binding affinity and hotspot for the lowest energy solutions found for polysorbate 20 and 80 solutions.

Molecule	P20	P20	P20	P20	P80	P80	P80
	1	2	3	4	1	2	3
*E*_binding_ kcal·mol^−1^	−21.3	−18.3	−15.6	−14.4	−20.6	−20.1	−18.9
Spot	2	3	1	1	2	3	1

**Table 5:** Description of the three hotspots identified for A33-based on the interaction with a set of commercial excipients.

Excipient	*E*_binding_ kcal·mol^−1^	Hotspot in Target
Trehalose_1	−13.8	**Hotspot 1** Heavy Chain: Lys43-Trp47; Arg67; Glu89 Light Chain: Ile1-Trp5; Thr97-Gln100
Trehalose_2	−12.3	
Sucrose_1	−16.9	
Sucrose_2	−13.9	
Arginine_1	−22.7	**Hotspot 2** Heavy Chain: Ser131-Ser136 Light Chain: Ala112-Ile117; Leu201-Asn210; Glu213-Cys214
Glycine_1	−27.3	
Glycine_2	−25.3	
Mannitol_1	−11.9	
Mannitol_2	−10.7	
Mannitol_3	−7.8	
Sorbitol_1	−9.4	
Sorbitol_2	−8.9	
Sorbitol_3	−8.5	
Tween20_1	−14.4	**Hotspot 3** Heavy Chain: Val2-Leu4; Ala105-Trp107; Gln109 Light Chain: Lys39; Lys42-Thr46; His55-Val58; Pro80-Phe83; Gln166-Ser168
Tween20_2	−15.6	
Tween80_1	−18.9	

**Table 6:** Correlation between the three hotspots identified and the three slower energy solutions of the molecular docking for two A33 molecules.

	Solution 1	Solution 2	Solution 3
Chain with higher number of interactions	Heavy	Light	Light
Matching hotspots	3 (light and heavy chain)	1 (light and heavy chain) and 2 (light chain)	1 (light and heavy chain)

**Table 7:** *T*_m_ values determined for Fab A33 with and without excipients. All experiments were performed in 10 mM phosphate buffer, pH 7; the average standard deviation was 0.28. Average binding affinity values from docking experiments above.

Sample	Concentration	*T*_m_ (°C)	Average *E*_binding_ kcal·mol^−1^
A33	1 mg/mL	79.37	-
A33 + trehalose	5% *w*/*v*	80.4	−13.05
A33 + sucrose	5% *w*/*v*	80.16	−15.4
A33 + mannitol	4% *w*/*v*	80.06	−10.13
A33 + sorbitol	4% *w*/*v*	80.3	−8.93
A33 + Tween 20	0.4% *w*/*v*	80.5	−15.00
A33 + Tween 80	0.4% *w*/*v*	80.1	−18.9
A33 + Gly	2% *w*/*v*	81.6	−26.3
A33 + Arg	2% *w*/*v*	71.6	−22.7

## Abbreviations

MDPI	Multidisciplinary Digital Publishing Institute
DOAJ	directory of open access journals
TLA	three-letter acronym
LD	linear dichroism
